# Materials and Cavity Design Principles for Exciton-Polariton Condensates

**DOI:** 10.1021/acsnano.4c15929

**Published:** 2025-03-10

**Authors:** Martin Gomez-Dominguez, Evan J. Kumar, Katherine A. Koch, Ajay Ram Srimath Kandada, Juan-Pablo Correa-Baena

**Affiliations:** † School of Materials Science and Engineering, Georgia Institute of Technology, Atlanta, Georgia 30332, United States; ‡ Department of Physics and Center for Functional Materials, Wake Forest University, Winston-Salem, North Carolina 27109, United States; § School of Chemistry and Biochemistry, Georgia Institute of Technology, Atlanta, Georgia 30332, United States

**Keywords:** exciton-polariton condensation, 2D perovskites, semiconductors

## Abstract

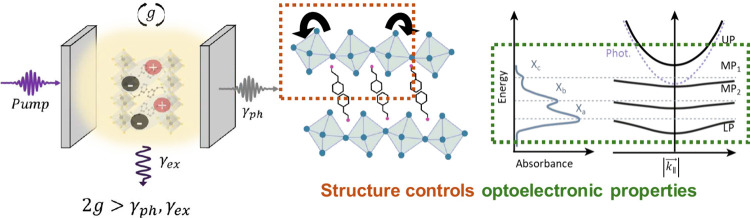

Exciton-polariton condensation offers a promising path to low-threshold coherent light sources, impacting fields from communications to healthcare. These hybrid quasiparticles, arising from strong exciton-photon coupling, combine the low effective mass from their photonic component and the strong nonlinear interactions from excitons. While polariton condensation has been achieved in a range of inorganic and organic materials, many systems still face significant challenges despite fulfilling the main properties requirements. In this perspective, we examine condensation mechanisms across different materials and highlight that universal guidelines do not exist; instead, we believe that exciton-polariton condensation is governed by the intrinsic properties of the active material. We propose using 2D perovskites as versatile platforms to investigate how specific structural and electronic characteristics influence the nonlinear processes driving exciton-polariton condensation. By exploiting the versatility of 2D perovskites, we can systematically explore and establish universal principles guiding the realization of polariton condensation in various material systems.

## 1 Introduction

Coherent light sources stand at the forefront of critical technological advancements: they power global communications, transform healthcare, and drive cutting-edge scientific research.^1^ The most ubiquitous form of coherent emission is lasing, which relies on population inversion to achieve macroscopic coherence. An analogous coherent light source is Bose–Einstein condensation (BEC), this is the macroscopic single state accumulation of bosons that occurs when the system reaches quantum degeneracy. This means that the de Broglie wavelength of a particle (λ_dB_) becomes comparable to the interparticle spacing, effectively when the density of bosons allows their wave functions to overlap.^2,3^ BEC, in contrast to lasing, does not require population inversion, which makes it an attractive mechanism to achieve low threshold coherent emission.^2^ Among the many systems that have shown transitions to Bose–Einstein condensates, microcavity exciton-polaritons are particularly promising.

**Figure 1:**
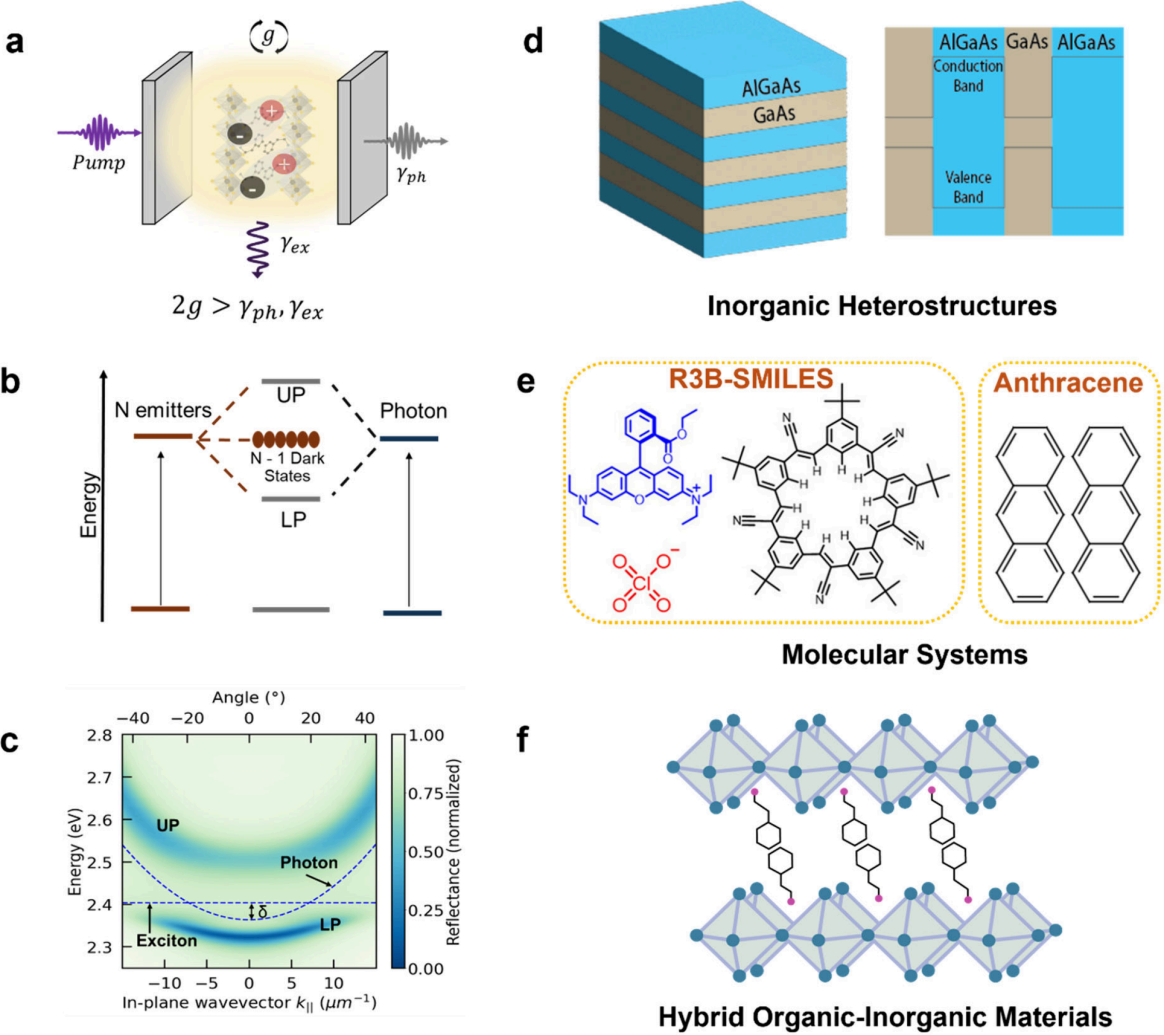
Engineering of exciton-polariton condensation. (a) Microcavity schematic in the strong light matter coupling regime, where γ_*ex*_ is the exciton recombination rate and γ_*ph*_ is the optical mode leakage rate. (b) N emitters interact strongly with photons to yield hybrid LP, UP and N – 1 dark states. (c) Characteristic angle dependent energy dispersion in the strong coupling regime displaying two emergent energy states: the lower and upper polariton (LP and UP), where δ is the detuning. The most common materials systems studied are inorganic (d), organic (e), and halide perovskites (f).

Microcavity exciton-polaritons are hybrid part-light part-matter quasiparticles that result from the near resonant, nondissipative energy exchange between excitons and modes of a confined electromagnetic field, in a regime known as strong light-matter coupling. These quasiparticles are produced in a microcavity (Figure 1a) by engineering an overlap of the photon mode in a microcavity and the absorption of the semiconductor. Here, the energy exchange between the material and cavity photon exceeds the population loss through exciton recombination (γ_*ex*_) or photon leakage (γ_*ph*_). Therefore, the energy of the system can no longer be described by distinct light and matter excitations but instead by two hybrid quasiparticles with inherited properties from both, the upper and lower exciton-polaritons (UP and LP, respectively).^1−3^ A molecular schematic of this scenario is depicted in Figure 1b. Due to their mixed light-matter nature, polaritons have different energies than their light and matter constituents and inherit properties from both: a low effective mass from light, and interactions from matter.

The signature of exciton polaritons is the anticrossing with a characteristic Rabi splitting (δ), which corresponds to the energy gap between polariton states at the in-plane wavevector in which the optical mode and the exciton are resonant. Figure 1c shows a common angle dependent energy dispersion of a strongly coupled system, the dashed parabolic line represents the cavity photon mode and the dashed horizontal line the energy of the exciton. When strongly coupled, these energies hybridize into the upper polariton (UP) and lower polariton (LP) modes. At different angles, or in-plane wavevectors , polariton states display different degrees of hybridization between light and matter. At higher wave vectors, the lower polariton dispersion is dominated by the exciton component with a flatter dispersion whereas at lower wave vectors, they exhibit a more pronounced parabolic dispersion. The lifetime of polaritons and the coupling strength are properties that depend, among other things, on the properties of the active layer coupling with light.

Bose–Einstein condensation occurs when a particle’s de Broglie wavelength exceeds the interparticle spacing. Since the de Broglie wavelength scales as λ_dB_ ∝ , where *m* is the particle mass and *T* is the temperature, the very small effective mass of polaritons results in a relatively large de Broglie wavelength.^4^ This property allows them to reach quantum degeneracy even at room temperatures. Furthermore, the ability to interact given by their matter constituent allows polaritons to make nonlinear interactions that aid the relaxation processes to the ground state, ultimately leading to condensation.

Exciton- polariton condensation was demonstrated over two decades ago,^5,6^ since then a wide variety of materials have managed to achieve polariton condensation. From inorganic semiconductor quantum wells, such as GaAs,^7^ hosting Wannier-Mott excitons (Figure 1d), to organic molecular systems like R3B-SMILES^8^ and anthracene^4^ with molecular excitations or Frenkel excitons (Figure 1e), enormous progress has been made toward achieving both low and room-temperature polariton condensation. In general, the sought-after materials requirements for polariton condensation include narrow line widths, strong exciton binding energies and large oscillator strengths.^9^ However, there are several other often unknown material parameters that drive the nonlinearities of the systems and govern the condensation process. While most work on exciton-polariton condensation has been on inorganic and organic materials systems, halide perovskites (Figure 1f) have emerged as an important materials system, due to their impressive optoelectronic properties.^10^ This perspective will focus on providing a framework to design new materials to achieve exciton-polariton condensation. We will explore how halide perovskites can be used to understand the role of materials parameters, such as structure, to understand exciton-polariton condensation.

## 2 Mechanisms of the Condensation Processes

In the generalized Tavis-Cummings picture of strong light-matter coupling, coupling of an ensemble of excited molecular states will result in a background density of dark states, referred to as the reservoir, in addition to the lower and upper polariton branches.^6,11,12^ Polariton condensation in this picture is fundamentally driven by dynamic processes that funnel the photoexcited population from the reservoir and the higher energy polariton states into the  point of the lower polariton branch.

Under nonresonant excitation, much of the photogenerated population lies within the exciton reservoir. Radiative pumping processes through appropriate cavity-detuning are exploited to effectively transfer the population into the lower polariton state, which then relaxes to the k = 0 state to stimulate the formation of the condensate. This photophysical mechanism is typically captured using a semiclassical kinetic model.^13−16^ which considers Bosonic stimulation of population transfer from the reservoir to the condensate, whose decay is determined by the polariton and reservoir lifetimes. In this picture the dynamics of the population occupation of the  point in the lower polariton dispersion, *n*_*p*_(*t*) can be described by^14^
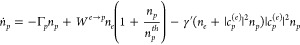
1

While this kinetic equation is widely used to model the time-resolved dynamics of polaritons, it is less descriptive than a Gross-Pitaevskii simulation, or a quantum dynamics model based on the Tavis-Cummings Hamiltonian. However, it offers a straightforward and intuitive approach to evaluating the key physical parameters that influence condensation thresholds. The above equation has three independent contributions. The first term is associated with the decay of the polariton at a rate Γ_*p*_, which is in turn determined by the cavity lifetime and the exciton lifetime, both related to material and layer quality. In emerging cavity architectures based on solution processing methods the polariton lifetimes are essentially limited by the cavity lifetimes in the picosecond or even subpicosecond time ranges with comparatively low quality factors associated with the cavities due to macroscopy defects. For an effective condensation at reasonably low excitation thresholds, the polariton lifetime must be substantial enough to allow sufficient population density of polaritons, above the threshold density *n*_*p*_^*th*^ to occupy the *k* = 0  state.

In a typical excitation scheme, however, the polariton states are populated through nonresonant excitation of the exciton reservoir. Even under resonant excitation conditions, a substantial fraction of photoexcitations inevitably occupy the reservoir states.^6^ Accordingly, the primary process in ensuring the accumulation of population in the LP state is the transfer of population from the exciton reservoir into the LP state, characterized by *W*^*e*→*p*^ in the equation above and *n*_*e*_ the population of excitons in the reservoir. Notably, the photophysical dynamics measured through time-resolved spectroscopies are dominantly determined by the exciton dynamics, which are driven by the exciton decay and nonlinear exciton–exciton quenching processes, all happening within the reservoir.^16,17^ In the weak coupling limit, the transfer dynamic scales with the exciton population. The additional term with the ratio  in the equation is the stimulated scattering of exciton population into the LP state. It must be noted that this scenario is very equivalent to photonic lasing driven by optical gain of the material.^18,19^

**Figure 2:**
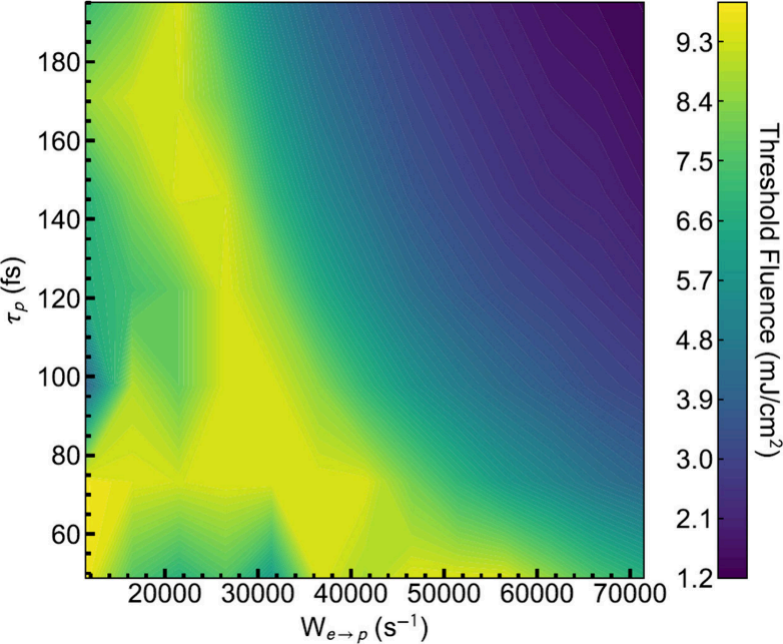
Threshold fluence for a nonresonant pump into the exciton reservoir estimated using a kinetic model described by eq 1 (and the associated coupled equation for the exciton population), plotted as a function of the polariton lifetime and the transfer rate from the reservoir to the lower polariton state.

Considering the first two terms in the equation along with its coupled counterpart associated with the dynamics of the reservoir and ignoring the polariton thermalization via nonlinear scattering processes (third term in the equation), we can estimate the excitation threshold density for typical values of polariton lifetime (τ_*p*_) and the transfer rate (*W*^*e*→*p*^). As can be visualized in Figure 2, the threshold excitation fluence is substantially lowered at larger polariton lifetimes and shorter transfer rates. Effective transfer of population from the reservoir and longer lifetimes of polaritons ensure that the critical density for condensation is achieved for the Bosonic stimulation of the condensate at lower excitation densities.

As noted earlier, the polariton lifetimes are fundamentally limited by the quality factors of microcavities. The cavities for inorganic quantum wells are accordingly carefully designed to have lifetimes in the order of tens to hundreds of picoseconds. This is also possible due to the high-quality epitaxial fabrication route employed for that purpose. Emerging material systems, including organic and hybrid semiconductors, however, are often self-assembled from solutions and it is challenging to obtain high Q-factor cavities embodying these materials. This can substantially increase the threshold densities in such cavities to levels that are physically impossible to generate without material degradation. Alternatively, a faster transfer rate may offer a way to reduce the threshold density.

**Figure 3:**
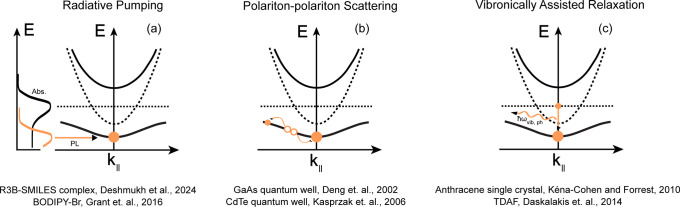
Schematic representation of (a) radiative pumping, (b) polariton-polariton scattering and vibronically assisted relaxation processes that populate the *k* = 0 state in the lower polariton dispersion and subsequently initiating the condensation process. Below each schematic are the reported examples of condensation in material systems, where such relaxation processes have been invoked. Refer to refs (8,28−32, and 56).

The physical processes that are embedded within the phenomenological parameter *W*^*e*→*p*^ are however more complex in nature. One widely proposed mechanism involves radiative pumping of the lower polaritons.^20,21^ Under appropriate design considerations, when the energy of the emission from the reservoir overlaps with the energy of the lowest LP state, the population from the reservoir can be effectively transferred to the LP state via emission-reabsorption processes, see the schematic in Figure 3a. Notably, due to the finite spectral line widths of excitonic transitions within the reservoir, such a transfer inevitably results in the occupation of portions of the lower polariton dispersion around the zero-momentum point. Additional thermalization mechanisms are needed to effectively funnel the population into the  state to further reduce the threshold density.

Momentum-conserving scattering processes are invoked to that purpose. At larger k-points, depending on their excitonic fraction, the polaritons can undergo many-body scattering driven by the excitonic Coulomb interactions. Polariton-polariton scattering, schematically shown in Figure 3b can be effectively described by the third term in the equation and γ′ parameter. This leads to the transfer of a polariton into the  state while transporting another to an even higher-lying state. Such an interaction is fundamentally governed by the underlying excitonic character of the polariton,^20,22−24^ as also quantitatively perceived through the Hopfield coefficients *c*_*p*_^(*e*)^ in the equation. Stronger excitonic interactions can accordingly aid the parametric scattering of polaritons. However, such interactions can also drive the nonlinear scattering of polaritons with the exciton reservoir and the nonlinear quenching processes within the reservoir, both of which are detrimental to the overall polariton population. Close to the bottom of the lower polariton dispersion interactions of polaritons with acoustic and optical phonons may be employed to further bring down the population to the *k* = 0  state.^21,25,26^ In some material systems, presence of vibronic excited states within the reservoir, schematically shown in Figure 3c can further aid the polariton thermalization dynamics.^27^ We highlight that polariton thermalization, via polariton-polariton scattering or vibronic relaxation processes, could play a more significant role than the radiative pumping mechanism, which is not necessarily a prerequisite for their manifestation, and the overall scenario is strongly material-dependent.

Even in near-ideal material systems, radiative pumping transfers the population from the reservoir to a relatively broad range of states within the lower polariton dispersion. For condensation to occur, it is crucial that the population converges into the k = 0 state, which relies on many-body interactions. These interactions differ across material systems, leading to bottleneck effects where the population fails to fully relax to the lowest energy state in the dispersion.

While there exists a wealth of literature on several photophysical mechanisms driving the relaxation of the polariton population into the zero-momentum state, material variables that can be tuned to optimize this rate are yet to be rigorously identified. In fact, all the relaxation processes described above and sketched in Figure 3a-c have been invoked in a variety of material systems. However, a distinct classification of materials based on the relaxation processes cannot be made. This must be pursued on a case-to-case basis and no universal principle may be derived for all material systems.

## 3 Polariton Condensation in Common Materials Systems

### 3.1 Inorganic Semiconductors

After Imamoglu’s theoretical proposal of polariton condensation,^33^ the first experimental claim using GaAs quantum wells (Figure 1d) showed a buildup of coherence and line width narrowing above a threshold, initially interpreted as condensation. However, it soon became clear that this was photonic lasing rather than true exciton-polariton condensation. This gave rise to materials challenges, as exciton bleaching at high densities shifted the system out of the strong coupling regime, preventing true condensation.^34,35^ Subsequent studies confirmed these findings, describing the saturation of the exciton states within the quantum well at high excitation densities that drove the system out of strong coupling.^36,37^ Furthermore, Tassone et al. emphasized the need for improved materials to address these challenges to condensation, describing bottleneck effects in the photoluminescence of GaAs microcavity polaritons, where slow polariton relaxation near the bottom of the dispersion curve limited condensation, despite an effective radiative pumping process enabling the population to reach the polariton states.^38^ By identifying the fundamental materials limitations in previous cavity designs that led to exciton saturation, later work addressed these challenges by designing a microcavity with 12 GaAs quantum wells. This design reduced exciton saturation effects and maintained strong coupling through improved Rabi splitting.^29^ In this configuration, with improved polariton lifetime, polariton-polariton scattering emerged as the primary relaxation mechanism for condensation, highlighting parametric scattering as a key process.^5,39,40^

Building on this approach, Kasprzak et al.^41^ demonstrated polariton condensation using CdTe/CdMgTe quantum wells. The higher exciton binding energy in CdTe (∼10 meV) compared to GaAs (around 4 meV) helped overcome the bottleneck effect by increasing exciton stability at high densities, thereby enabling efficient polariton relaxation toward the ground state.^42^ Additionally, the use of a 16 quantum well cavity, enhanced the Rabi splitting to 26 meV and enabled stronger polariton-polariton interactions.^41^ This combination of material choice, increased quantum well count, and controlled cavity conditions facilitated efficient polariton scattering and relaxation into the ground state. Second-order coherence measurements in this setup confirmed the formation of a quantum condensate, emphasizing that material quality, quantum well design, and carefully managed coupling conditions are instrumental to achieving polariton coherence and condensation. Moreover, CdTe microcavities have been used to investigate the effect of cavity detuning on condensation. In a follow-up study, Kasprzak et al.^43^ performed experiments at different detunings and found that negative detuning leads to condensation driven predominantly by kinetic, nonthermal relaxation, whereas positive detuning supports a more thermalized, thermodynamically governed process. When the detuning results in an exciton fraction larger than the photon fraction, the reduced cooling time and extended lifetime favor thermalization. Increasing the photonic component, however, shifts the condensate into a more distinctly nonequilibrium regime. Complementary work by Richard et al.^44,45^ and Bajoni et al.^44^ further reinforces that careful detuning is a key factor for preserving strong coupling and optimizing polariton relaxation mechanisms.Despite this system’s non linearities being governed primarily by polariton-polariton parametric interactions, recent work by Emma et al.^45^ has highlighted the significant influence of the ensemble of excitonic states that remain uncoupled or weakly coupled to light, known as the exciton reservoir. This work reveals that characteristics previously attributed solely to parametric scattering—such as emission broadening and blueshift near the condensation threshold—are also affected by interactions with the exciton reservoir.^45^ Understanding the role of the exciton reservoir deepens our insight into polariton condensation mechanisms and has significant implications for material systems with large excitonic nonlinearities.

### 3.2 Organic Semiconductors

The transition to organic semiconductors for polariton condensation revealed the challenges to achieve condensation across materials systems. The understanding that polaritons, being quasiparticles, have complex interactions that depend on the nature of their crystal ground state is particularly relevant in this context. Systems hosting Wannier-Mott excitons, which have a large spatial extent, facilitate significant exciton–exciton overlap and stronger exciton–exciton interactions.^46−48^ In organic microcavities, polariton condensation faces significant challenges due to the material properties of organic molecules and crystals, such as additional decay pathways for annihilation of excitons.^49,50^ high vibronic coupling,^51,52^ and energetic and structural disorder.^53,54^ Furthermore, due to lack of structural control in the fabrication processes in organic materials, there is little control of the molecular and crystallographic orientation in these thin films. This can lead to misalignment in the dipole moment orientation that reduces the ability to couple to a given electric field polarization, resulting in reduced Rabi splitting.^4^ All these factors complicate polariton relaxation and lead to rapid nonradiative decay, which hinders the formation of polariton condensates.^55^

Given the differences in the material environment hosting exciton-polaritons and the unique properties of excitons in these systems, condensation could not follow the same approach as in inorganic cavities. The nonlinear relaxation processes in organic systems are distinct, requiring new strategies tailored to the characteristics of organic materials. The first demonstration of polariton condensation in an organic material was achieved using single crystals, which were thought to minimize disorder compared to thin films, thereby improving Rabi splitting and polariton relaxation. Kéna-Cohen and Forrest used anthracene single crystals in their microcavity design, taking advantage of the high exciton binding energy and the ordered structure of anthracene to lower the condensation threshold.^56^

Despite these favorable qualities, previous studies in anthracene systems had not achieved condensation but had observed intermediate polariton states due to hybridization with vibronic transitions.^57^ Litinskaya et al. proposed that excitons in the reservoir could relax to polariton states through nonradiative decay by emitting intramolecular vibrations.^58^ Kéna-Cohen and Forrest confirmed this for these anthracene systems by designing microcavities with precise detuning, demonstrating that condensation occurred only when the minimum energy of the lower polariton branch was positioned exactly one vibrational energy below the zero-phonon line energy. This alignment allowed direct population of the polariton ground state, rather than through multistep relaxation, favoring the specific characteristics of this organic system. It was therefore possible to achieve macroscopic coherence in these systems by understanding material properties of anthracene and their effect on the nonlinearities in the strong light matter coupling regime.

After the pioneering work of Kéna-Cohen and Forrest with anthracene single crystals, the field has rapidly expanded to include a broader range of organic materials such as fluorescent proteins, organic dyes, and oligomers that host Frenkel excitons. These materials not only allow for robust room-temperature polariton condensation but also exhibit pronounced nonlinear phenomena and enhanced polariton-polariton interactions. For example, Wei et al.^59^ recently demonstrated low-threshold room-temperature polariton lasing in fluorene-based oligomers by exploiting the interplay between exciton reservoir dynamics and vibronic transitions, highlighting the importance of nonlinear effects in these systems. In a related study, Grant et al.^60^ showed that efficient radiative pumping in strongly coupled microcavities containing fluorescent dyes can significantly aid the population of lower polariton states, emphasizing the role of fast radiative rates and high photoluminescence quantum yields for systems where polariton relaxation is governed by radiative pumping. Complementary experimental and theoretical studies including recent reports^63−65^ have enriched the discussion on nonlinear effects in organic exciton-polariton systems with localized Frenkel excitons.

### 3.3 Hybrid Organic–Inorganic Perovskites

The first demonstration of room-temperature polariton condensation in lead-halide perovskites was achieved using all-inorganic CsPbCl_3_ within a planar microcavity.^56^ This system exhibited strong exciton-photon coupling, with a Rabi splitting of 270 meV. Similar behavior has been observed in CsPbBr,^61−63^ where high exciton binding energies (40–75 meV)^64^ and clean absorption spectra make CsPbCl_3_ and CsPbBr_3_ promising candidates for polariton condensation.^65^ In contrast, bulk perovskites with organic cations like MAPbBr_3_ and FAPbBr_3_ have lower exciton binding energies (14–25 meV),^64^ making room-temperature exciton stability more challenging and reducing polariton lifetimes. This makes inorganic cation perovskites, like CsPbBr_3_ and CsPbCl_3_, more suitable 3D perovskites for polariton condensation.

Two-dimensional (2D) perovskites, with large exciton binding energies (∼400 meV^66−68^) and strong oscillator strengths, are ideal candidates for polariton condensation. However, room-temperature polariton condensation in these materials remains elusive. To date, there is only one condensation report in 2D perovskites at low temperatures.^28^ Recent research on energy and momentum exchange mechanisms in exciton-polariton systems in 2D perovskites has identified a strong polariton bottleneck that prevents relaxation to smaller wave vectors states.^69^ This bottleneck decreases at temperatures below 60 K, where the excitonic emission spectrum sharpens, highlighting that polariton relaxation involves the exciton reservoir, which consists of states uncoupled or weakly coupled to light.

In our recent work, we investigated the role of the exciton reservoir in these pure two-dimensional materials, proposing that the complex scattering landscape between the exciton reservoir and polaritons limits polariton condensate formation.^70^ Moreover, we identified multiple radiative pathways in 2D perovskites that inefficiently pump the lower polariton branch at its lowest energy state.^71^ These pathways arise from strong polaronic effects, where excitons couple to distinct lattice vibrations, creating nondegenerate excitonic states with varying degrees of polaronic character.^72^ This multiplicity of resonances adds additional polaritonic states, complicating efficient scattering into the polariton ground state, a crucial requirement for condensation.

**Figure 4:**
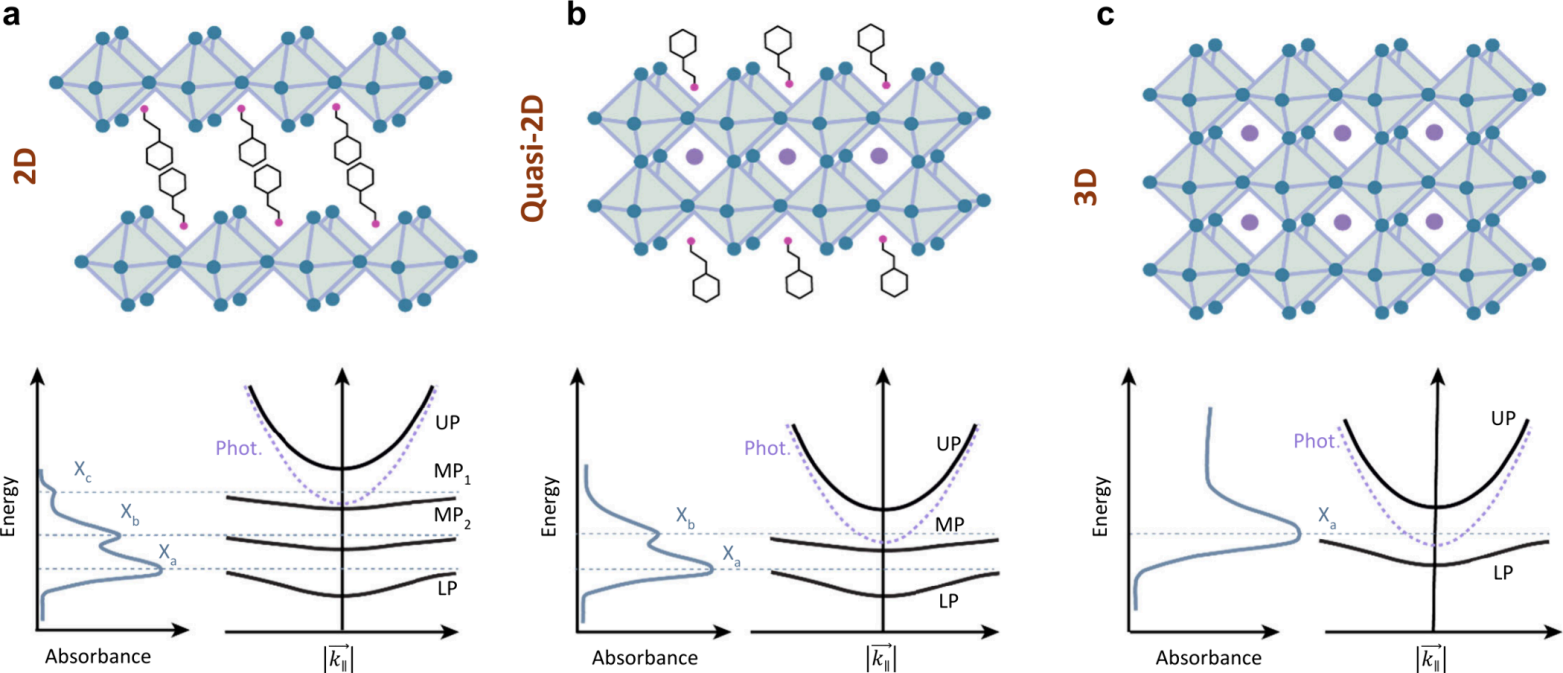
Polariton condensation roadblocks in halide perovskites as a function of structural dimensionality. (a) 2D *n* = 1 halide perovskites, (b) mixed dimensional materials where more than one (*n* > 1) inorganic slabs are separated by bulky cations, and (c) traditional 3D perovskites with no bulky cations involved. The proposed processes for polariton formation are depicted below the structures.

It must be highlighted that the multiplicity in excitonic resonances ubiquitous in 2D systems is notably absent in the case of 3D perovskites, as depicted in Figure 4. Accordingly, as sketched in Figure 4, this results in a much simpler polariton dispersion with more straightforward relaxation scheme. Appropriate control of the dimensionality may enable one to obtain a more suitable excited state scheme, with single excitonic transition yet with large oscillator strength and narrow line width. Inherent to this problem is the question of the origin of the fine structure, which is yet to have a clear answer. Additionally, as more layers of inorganic octahedra are incorporated between organic ligands, the spatial extent of the excitons increases. A larger exciton Bohr radius enhances nonlinear exciton–exciton interactions, which facilitates polariton relaxation processes and enables macroscopic ground state polariton accumulation.^73^

### 3.4 Path Forward for Halide Perovskite Polaritons

Enhancing the cavity quality factor is a well-established and effective approach to lowering the condensation threshold. However, solution-processed materials inherently face challenges in being embedded within high-Q cavities. While a high-Q factor is undeniably crucial, achieving optimal condensation in a strongly coupled system also requires careful consideration of various material parameters. Polaritons inherently interact not only with each other but also with the unique crystal ground state of the material. Understanding the nonlinear relaxation processes occurring in the strong light-matter coupling regime requires some baseline understanding of the material system itself and is crucial for designing semiconductor microcavities that support final-state stimulation and efficient polariton condensation.

**Figure 5:**
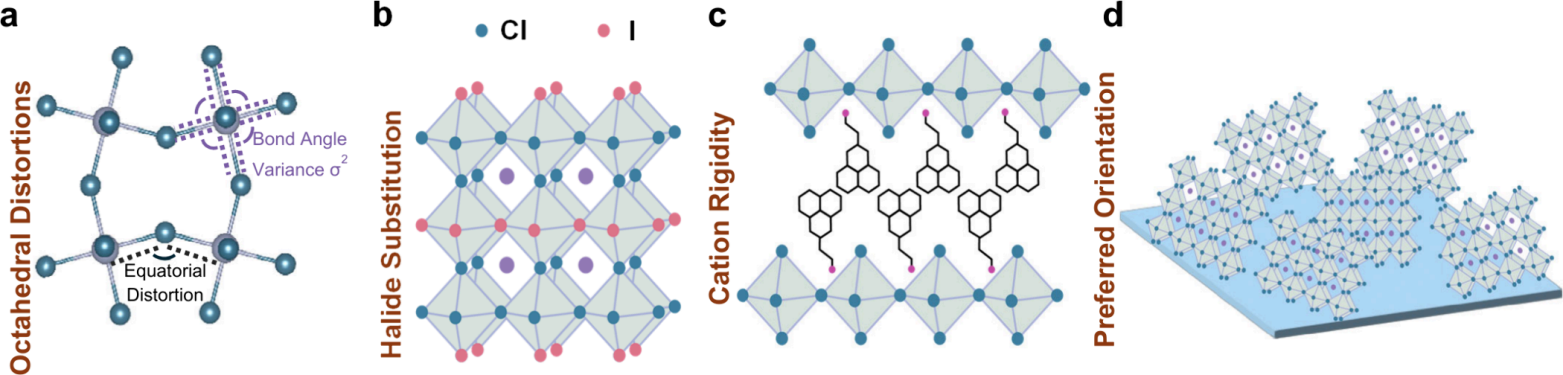
Structural handles to modify optoelectronic properties in halide perovskites. (a) A-site cation interactions with the lead halide octahedra enables control over their distortions. (b) Chemical manipulation enables bandgap, bond length, and octahedral tilt control. (c) A-site cation chemistries enable their rigidity in between the lead halide sheets. (d) Materials processing can be used to control preferred crystallographic orientation.

Hybrid organic–inorganic 2D perovskites are particularly promising in this context due to their ideal material properties and the ease with which these properties can be tuned. They can be grown as high-quality single crystals, allowing precise structural characterization.^74,75^ The myriad of structural characteristics of perovskites can serve as material handles for achieving condensation. For instance, through interactions of the organic cation the Pb–I–Pb bond angle can be altered as seen in Figure 5a, this modifies the electronic band structure, a smaller Pb–I–Pb bond angle (from increased octahedral tilting) widens the band gap by reducing Pb orbital overlap.^76^ Additionally, octahedral distortions can be also induced by changing the organic cation and these have shown to have an effect in the spectral structure of the material.^77^ Furthermore, the electronic structure is primarily determined by the halide-lead bond, choosing different halides—as seen in Figure 5b—(I, Br, Cl) directly affects the band structure and exciton energies,^78,79^ enabling precise tuning of electronic properties to favor condensation,^85^ Moreover, the organic spacer cations modify the lattice dynamics, affecting exciton–phonon interactions,^80−82^ adjusting these interactions, by controlling the rigidity of the overall structure (Figure 5c), can help support the nonlinear dynamics necessary for condensation. Finally, the crystallographic orientation of the perovskite (Figure 5d) structure can induce anisotropy in the excitonic properties,^83−85^ which can be exploited to enhance polariton coherence and condensation dynamics.^86^

These tunable properties summarized in Figure 5 enable the development of material-specific guidelines to achieve polariton condensation. For example, in materials with low exciton binding energies, strategies might focus on enhancing dielectric confinement to increase binding energy. In systems with strong phonon interactions, designing the lattice to suppress specific phonon modes could reduce exciton scattering and promote condensation. By leveraging the versatility of 2D perovskites, we can systematically explore and establish universal principles guiding the realization of polariton condensation in various material systems.

## 4 Conclusions

We have provided a comprehensive overview of the key material factors in inorganic, organic, and hybrid semiconductors that can be strategically engineered to achieve room-temperature and low-threshold polariton condensation. Beyond their role as low-threshold lasers, polariton condensates have emerged as powerful platforms for quantum information science. Their strong nonlinearities and unique many-body interactions make them particularly well-suited for quantum simulation, enabling the exploration of complex many-body Hamiltonians and lattice models. Additionally, their inherent coherence makes them valuable for high-sensitivity interferometric schemes, advancing precision metrology. While polariton condensates have a wide range of potential applications, an exhaustive discussion is beyond the scope of this work; we instead direct readers to existing reviews for a more detailed exploration. The limiting factors discussed in this perspective play a crucial role in the design of optimized material architectures, ensuring that polariton condensates can be fully harnessed across these diverse applications.
